# Curcumin Reverses the Diazepam-Induced Cognitive Impairment by Modulation of Oxidative Stress and ERK 1/2/NF-*κ*B Pathway in Brain

**DOI:** 10.1155/2017/3037876

**Published:** 2017-10-02

**Authors:** Alexandra C. Sevastre-Berghian, Vlad Făgărăsan, Vlad A. Toma, Ioana Bâldea, Diana Olteanu, Remus Moldovan, Nicoleta Decea, Gabriela A. Filip, Simona V. Clichici

**Affiliations:** ^1^Department of Physiology, “Iuliu Hatieganu” University of Medicine and Pharmacy, 1 Clinicilor Street, 400006 Cluj-Napoca, Romania; ^2^Department of Molecular Biology and Biotechnology, Faculty of Biology and Geology, Babes-Bolyai University, Clinicilor Street No. 5-7, Cluj-Napoca, Romania; ^3^Department of Biochemistry and Experimental Biology, Institute of Biological Research, Republicii Street No. 48, branch of NIRDBS Bucharest, Cluj-Napoca, Romania; ^4^Department of Molecular and Biomolecular Physics, NIRD for Isotopic and Molecular Technologies, Donat Street No. 101, Cluj-Napoca, Romania

## Abstract

Oxidative stress and inflammation can be involved in cognitive dysfunction associated with neurodegenerative disorders. Diazepam (DZP) administration has been chosen to simulate the memory impairment. The aim of this study was to evaluate the effects of curcumin (CUR) on spatial cognition, ambulatory activity, and blood and brain oxidative stress levels. The ERK/NF-*κ*B signaling pathway and the histopathological changes in the hippocampus and frontal lobe, in diazepam-treated rats, were also analyzed. The animals were divided into 4 groups: control, carboxymethylcellulose (CMC) + CUR, CMC + DZP, and CUR + CMC + DZP. CUR (150 mg/kg b.w.) was orally administered for 28 days. DZP (2 mg/kg b.w.) was intraperitoneally administered 20 minutes before the behavioral tests (open field test, Y-maze, and elevated plus maze). CUR improved the spontaneous alternation behavior, decreased the oxidative stress levels, both in the blood and in the hippocampus, and downregulated the extracellular signal-regulated kinase (ERK 1/2)/nuclear transcription factor- (NF-) *κ*B/pNF-*κ*B pathway in the hippocampus and the iNOS expression in the hippocampus and frontal lobe of the DZP-treated rats. Histopathologically, no microscopic changes were found. The immunohistochemical signal of iNOS decreased in the DZP and CUR-treated group. Thus, our findings suggest that curcumin administration may improve the cognitive performance and may also have an antioxidant effect.

## 1. Introduction

Memory, one of the most complex brain functions, involving multiple neuronal pathways and neurotransmitters, is considered the ability of an individual to record, retain, and recall the information when needed [1, 2].

Aging, stressful conditions, reduced brain metabolism, high oxidative stress levels, inflammation, or reduced plasticity has been hypothesized to be involved in cognitive dysfunction associated with neurodegenerative disorders, such as Alzheimer's (AD) or Parkinson's disease (PD) [3–5]. The high incidence of the abovementioned disorders over the past decades determined the scientists' focus on different therapies in order to improve the quality of life of these individuals suffering from neurodegenerative diseases. Although, in the past years, the stem cell therapies have been tried to attenuate the progression of these disorders, none of them has fully proved its efficiency [6].

Considering the multiple hypotheses regarding the mechanisms that lead to neuronal dysfunction, for example, inflammation [7], oxidative stress [8], mitochondrial dysfunction [9], and axonal transport deficits [10], the need of an alternative therapy that may provide some symptomatic relief is highly needed [3–5, 11]. However, neuroprotection does not seem to fully inhibit the disease progression, at least a delay can be achieved.

Curcumin (CUR), a natural compound (1,7-bis(4-hydroxy-3-methoxyphenyl)-1,6-heptadiene- 3,5-dione), found as a major component in turmeric, extracted from the rhizome of *Curcuma longa L*., has been used in traditional medicine for thousands of years. Nowadays, it is widely used in South and Southeast Asia as a spice and as a coloring agent in food. In the past years, several scientific reports have mentioned its potential benefits, some of which are anticarcinogenic, neuroprotective, anti-inflammatory, and antioxidant effects [12–14]. At molecular level, curcumin downregulates various proinflammatory intracellular systems such as NF-*κ*B, inducible nitric oxide synthase (iNOS), hypoxia-inducible factor-1, and proinflammatory cytokine such as: interleukin- (IL-) 6, IL-1*β*, and tumor necrosis factor- (TNF-) *α*. It also exerts antiapoptotic function by overexpression of Bcl-2 and decrease of the bax/Bcl-2 ratio. As an antioxidant, curcumin increases Cu-Zn superoxide dismutase (SOD), restores depletion of the glutathione (GSH) levels, inhibits ROS and mitochondrial cell death pathway, and activates the nuclear factor erythroid 2-related factor 2/antioxidant responsive element (Nrf2/ARE) pathway [6, 12].

As curcumin has been suggested to prove valuable properties, the present study was designed to investigate the effects of curcumin administration on ambulatory activity and spatial working memory (a form of short-term memory) and on blood and brain oxidative stress levels in diazepam-treated rats. Based on the literature, 2 mg/kg body weight (kg b.w.) of diazepam would impair memory in rodents [15]. The administration of diazepam (DZP, 7-chloro-1,3-dihydro-1-methyl-5-phenyl-2H-1,4-benzodiazepin-2-one), a benzodiazepine compound, serves as an interoceptive behavioral model (the stimulus lies within the body) to evaluate memory in rodents [1, 3, 16, 17].

Benzodiazepines (BZDs) are widely used as anxiolytics, sedative hypnotics, anticonvulsants, and muscle relaxants or for anesthesia induction. Although BZDs are well tolerated, they are still known for their clinical issues such as cognitive and psychomotor impairment, sedation, dependence, rebound anxiety, and discontinuation syndrome [18–21]. Memory seems to be particularly sensitive to BZD action, known as “acquisition impairing” molecules [22]. Thus, BZDs impair long-term memory, more specifically anterograde memory (amnesia for events occurring after drug absorption, for instance) [18, 23].

The target of BZDs is the *γ*-aminobutyric acid- (GABA-) A receptor, which is a ligand-gated chloride channel, activated by GABA. GABA is an amino acid that exerts an inhibitory neurotransmission in the central nervous system, thus reducing the excitability of neurons. [18].

The GABA-A receptor complex is composed of 5 glycoprotein subunits, containing 2 *α*, 2 *β*, and 1 *γ* subunits. Side effects of the BZDs, such as sedation and anterograde amnesia, are *α*1 mediated [22, 24]. Based on these data, our study intended to evaluate the effect of a high dose of DZP on spatial cognition in conjunction with oxidative stress, ERK 1/2/NF-*κ*B signaling pathway, and histopathological changes in the hippocampus, frontal lobe, and whole brain and the impact of curcumin pretreatment on these parameters in rats.

## 2. Materials and Methods

### 2.1. Reagents

Curcumin (CUR), Bradford reagent, trichloroacetic acid, and o-phthalaldehyde were obtained from Sigma-Aldrich Chemicals GmbH (Germany), and diazepam (DZP) (10 mg/2 mL) was purchased from Terapia Ranbaxy, Cluj-Napoca. 2-Thiobarbituric acid and EDTA-Na_2_ were obtained from Merck KGaA (Darmstadt, Germany) while absolute ethanol and n-butanol were from Chimopar (Bucharest, Romania). ELISA tests for total p44/42 MAP kinase (extracellular signal-regulated kinase (ERK) 1/2) were obtained from Cell Signaling Technology Inc. (Danvers, MA, USA), and antibodies against NF-*κ*B, pNF-*κ*B, and NOS2 (iNOS) and secondary HRP-linked antibodies were from Santa Cruz Biotechnology, Heidelberg, Germany. Curcumin was dissolved in 0.5 mL of carboxymethylcellulose (CMC).

All chemicals and reagents were of high-grade purity.

### 2.2. Animals and Experimental Design

Experimental procedures were approved by the Animal Ethics Board of “Iuliu Hatieganu” University on animal welfare according to the Directive 2010/63/EU on the protection of animals used for scientific purposes.

Two-month-old male Wistar rats (*n* = 40) were used under standard laboratory conditions, housed in a 12 h light–12 h dark cycle at room temperature (24 ± 2°C). The rats had free access to a standard normocaloric pellet diet and received water ad libitum.

**Figure 1:**
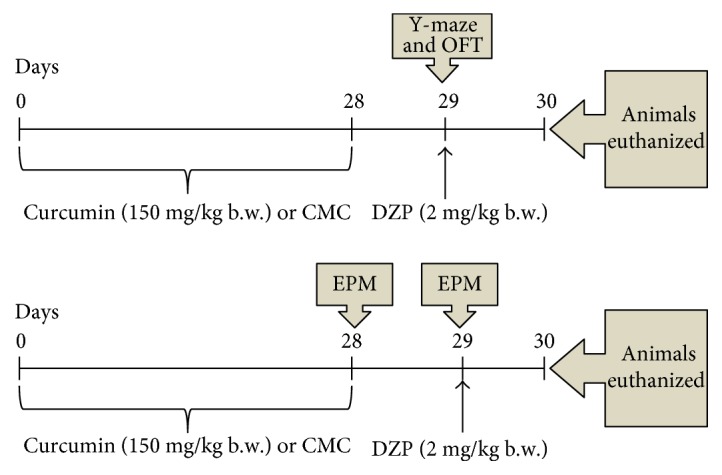
Experimental design. Four groups of animals were orally treated with 150 mg/kg b.w. curcumin for 28 days. Diazepam (2 mg/kg b.w.) was intraperitoneally injected 20 minutes before the behavioral tests. One day later, the blood, hippocampus, frontal lobe, and whole brain were harvested for biochemical and histopathological investigations. Tests—OFT: open field test; EPM: elevated plus maze; and Y-maze. DZP: diazepam.

To evaluate the effect of curcumin on ambulatory activity and spatial working memory and on oxidative stress in rats, induced by diazepam administration, the animals were divided into 4 groups of 10 rats each (Figure 1).

One group consisted of untreated rats and served as the control. The animals in the other 2 groups were given curcumin dissolved in carboxymethylcellulose (CUR + CMC) or carboxymethylcellulose and DZP (DZP + CMC). The last group of animals was treated with curcumin dissolved in CMC and with DZP (DZP + CUR + CMC). Curcumin was administered orally (150 mg/kg b.w. per day), dissolved in 0.5 mL CMC, for 28 days. The dose of 2 mg/kg b.w. of diazepam was intraperitoneally (i.p.) injected, 20 minutes before the behavioral tests were performed.

After four weeks of curcumin administration, the behavioral tests were conducted. Five rats out of ten, in each group, underwent the Y-maze and OFT test, while the other five rats were tested in the EPM in two consecutive days. Twenty-four hours after the last behavioral test, under anesthesia with an intraperitoneal injection of ketamine/xylazine cocktail (90 mg/kg b.w. ketamine and 10 mg/kg b.w. xylazine), all animals were euthanized. The blood and the whole brain tissues from 5 rats in each group were harvested for biochemical and histopathological investigations. From the other 5 rats in the group, the hippocampus and frontal lobe were harvested for the oxidative stress assays, ELISA test, and Western blotting technique.

### 2.3. Behavioral Testing

Three different tests were used in this particular study, such as the open field test (OFT) and elevated plus maze (EPM) to assess the general locomotor activity and anxiety-like behavior, Y-maze, for the spontaneous alternation behavior, and EPM to assess the transfer latency. The animals' movement was quantified by a visual tracking system (Smart Basic Software version 3.0 Panlab Harvard Apparatus) using specific mazes for rats (Ugo Basil Animal Mazes for Video-Tracking).

#### 2.3.1. Y-Maze Test

The Y-maze is a three-arm horizontal maze (10 × 50 × 20 cm) in which the arms are symmetrically separated at 120°. Rats were placed at the end of one arm of the maze and were allowed to freely explore the Y-maze for 8 minutes. Smart Software recorded and analyzed the sequence and number of arm entries based on animals' activity. An actual alternation was defined as entries into all three arms on consecutive choices. The data were presented as percentage of alternations. The percentage of alternations was defined according to the following equation: %alternation = [(number of alternations)/(total arm entries − 2)] × 100. Spontaneous alternation behavior is considered to reflect spatial working memory, which is a form of short-term memory [25, 26].

#### 2.3.2. OFT

Rats were placed in the center of an open field arena (100 × 100 × 40 cm) that was electronically divided into 9 equal squares. The animals were freely allowed to explore the apparatus for 5 minutes. Total and peripheral travelled distance and total and peripheral numbers of entries were considered as a general locomotion index. High central travelled distance and central number of entries and time ratio (central/total time) were considered as a low level of anxiety [27].

#### 2.3.3. EPM

The apparatus consists of a plus-shaped maze, with two open (10 × 50 cm) and two closed (10 × 50 × 40 cm) arms, elevated to 60 cm above ground level. On the 28th day of curcumin treatment, each rat was placed at the end of an open arm, facing away from the central platform. Transfer latency (TL) was defined as the time (in seconds) taken by the animal to move from the open arm into one of the closed arms. TL was recorded on the first day (training session) for each animal when the rat was allowed to explore the maze for 5 minutes. Retention of this learned task (memory) was examined 24 hours later [1, 3, 28, 29]. Diazepam was i.p. injected, 20 minutes before the EPM was performed, in the second day.

Measures of motor activity in this task are total and closed arm travelled distance and total and closed arm number of made entries. High open arm travelled distance and open arms number of entries and time ratio (open arms/total time) were considered as a low level of anxiety [30]. Between tasks, the mazes were cleaned with 70% ethanol to remove residual odor.

### 2.4. Biochemical Investigations

The malondialdehyde (MDA) levels were considered as lipid peroxidation markers, while the glutathione-reduced (GSH)/glutathione-oxidized (GSSG) ratio was interpreted as antioxidant biomarkers. The MDA levels in the blood, whole brain homogenates, hippocampus, and frontal lobe were determined by spectrofluorimetry, using the 2-thiobarbituric acid method. The values were expressed as nmol/mL and nmol/mg of protein [31]. GSH and GSSG were measured fluorimetrically using o-phtalaldehyde in the blood, whole brain homogenates, hippocampus, and frontal lobe homogenates. Their values were established using a standard curve and were presented in nmol/mL and nmol/mg protein or expressed as GSH/GSSG ratios [32].

### 2.5. Quantitative Estimation of ERK 1/2 Level and NOS2 and NF-*κ*B/pNF-*κ*B Expressions

Total p44/42 MAPK (ERK 1/2) protein level was evaluated by Path Scan Sandwich ELISA tests according to the manufacturer's protocol (Cell Signaling Technology Inc.). Results are expressed in terms of OD units/mg protein of different treatment samples. NF-*κ*B, phospho-NF-*κ*B, and iNOS quantification were performed by Western blot technique.

Lysates (20 *μ*g protein/lane) were separated by electrophoresis on 8% SDS PAGE gels under reducing conditions, then transferred to polyvinylidenedifluoride membranes (BioRad), using Biorad Miniprotean system (BioRad). Blots were then blocked and incubated with antibodies NOS2 (iNOS), NF-*κ*B, and phospho-NF-*κ*B (Ser536) (93H1) (Santa Cruz Biotechnology, Heidelberg, Germany) diluted in 1 : 500. After washing, the blots were incubated with corresponding secondary HRP-linked antibodies (1 : 1500) (Santa Cruz Biotechnology). Proteins were visualized and detected using Supersignal West Femto Chemiluminiscent substrate (Thermo Fisher Scientific, Rockford IL, USA) and a Gel Doc Imaging system equipped with a XRS camera and Quantity One analysis software (Biorad). GAPDH was used as a protein loading control.

### 2.6. Histological Investigation and Immunohistochemical Analysis of iNOS

At the end of the experiment, rats were euthanized. For histological analysis, brain samples were fixed in 10% neutral buffered formalin, then embedded in paraffin in order to produce 5 mm-thick sections which were stained with hematoxylin-eosin (HE) for light microscopy (Optika B-383LD2 microscope). For histopathological analysis, we observed the global hippocampal structures and frontal lobe.

For immunohistochemistry, brain samples were fixed in 4% paraformaldehyde in 0.1 M phosphate buffer at pH 7.4 for detecting NOS2 (iNOS). Samples were incubated overnight at 4°C with anti-NOS2 antibody (1 : 200; Santa Cruz Biotechnology, Santa Cruz, CA). Then, they were treated with an ABC kit (Dako). Chromogen substrate diaminobenzidine (DAB) was used. To check the specificity of the immunohistochemistry tests, tissues, in which primary antibodies were omitted from the initial incubation, were also prepared. For immunohistochemical analysis, we observed the CA3 field in the hippocampus and the frontal cortex.

### 2.7. Statistical Analysis

All statistical analyses were conducted using ANOVA GraphPad Prism software, version 6.0 (GraphPad, San Diego, California, USA). The results were expressed as the mean ± standard error of the mean (SEM). One-way analysis of variance (ANOVA) was used, followed by Tukey's post hoc test, to determine statistical significance among the four groups. Two-way analysis of variance (ANOVA) was used, followed by Bonferroni's post hoc test to determine statistical significance among the four groups for transfer latency parameter in the EPM. A *p* value lower than 0.05 was considered statistically significant. Results are expressed as follows: mean ± SEM; ^∗^*p* < 0.05 as compared with control/curcumin + CMC; and ^#^*p* < 0.05 as compared with DZP + CMC.

## 3. Results

### 3.1. Behavioral Studies

**Figure 2:**
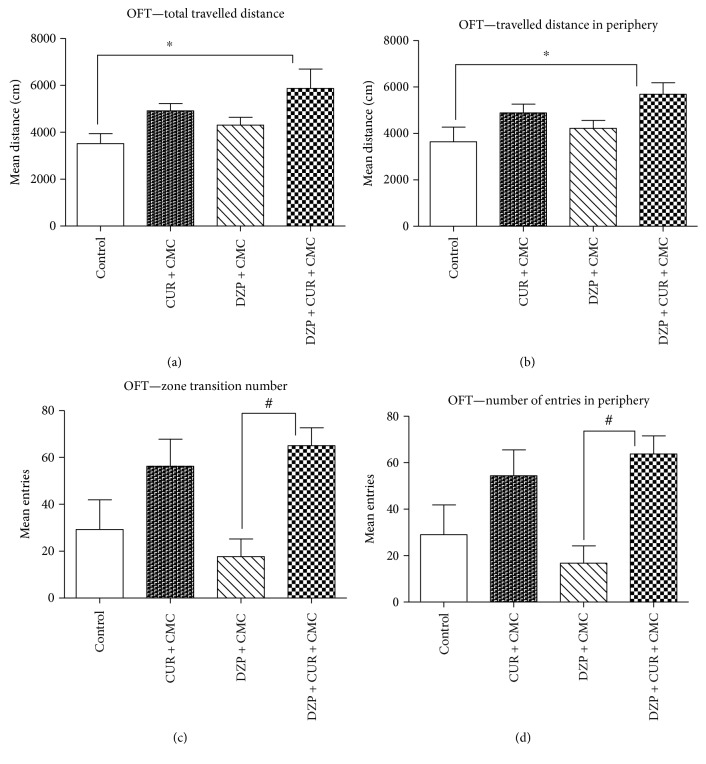
The effects of curcumin on total (a) and peripheral (b) travelled distance and total (c) and peripheral (d) number of entries in the open field test (OFT). Total travelled distance (a) and travelled distance in the periphery (b) were significantly increased in the diazepam and curcumin group as compared to the control group (*p* < 0.05). Four weeks of curcumin administration in comparison to DZP treatment increased significantly the locomotor activity (zone transition number (c) and number of entries in the periphery (d)) (*p* < 0.05). Each group consisted of 5 rats. Results are expressed as mean ± SEM. ^∗^*p* < 0.05 as compared with control/CUR + CMC; ^#^*p* < 0.05 as compared with DZP + CMC.

The effect of curcumin on rats' locomotion, tested in the OFT, is illustrated in Figure 2.

The OFT is a convenient method to measure both locomotor activity and anxiety-like behavior or sedation in rodents [30, 33, 34]. Our results showed that total travelled distance and travelled distance in the periphery were significantly increased in the diazepam and curcumin group as compared to those of the control group (*p* < 0.05). Four weeks of curcumin administration, in comparison to diazepam treatment, increased significantly the locomotor activity (zone transition number and number of entries in the periphery) (*p* < 0.05).

**Figure 3:**
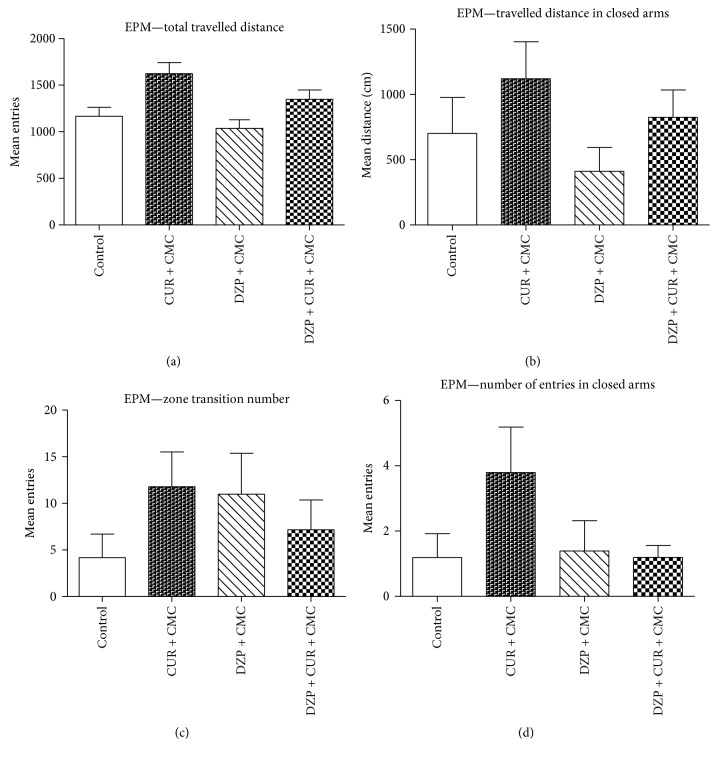
The effects of curcumin on the total (a) and peripheral (b) travelled distance and the total (c) and peripheral (d) number of entries in the elevated plus maze (EPM). The DZP-treated rats tended to travel less (a, b) and make fewer entries (c, d) (both as total and as closed arms scores), but the differences were not statistically significant (*p* > 0.05). Curcumin administration improved both the total travelled distance (a) and the distance in closed arms (b), but without any statistical significance (DZP + CUR + CMC versus DZP + CMC, *p* > 0.05). Each group consisted of 5 rats. Results are expressed as mean ± SEM.

The effect of curcumin on rats' locomotion, tested in the EPM, is illustrated in Figure 3. This task mainly provides measures for anxiety-like behavior, but the locomotor activity can be recorded, as well [30]. In the EPM, there was no significant effect of diazepam as compared to the curcumin group on general locomotion (total and peripheral travelled distance and total and peripheral number of entries) (*p* > 0.05). Curcumin administration tended to improve both total travelled distance and distance in closed arms, but without any statistical significance (*p* > 0.05).

**Figure 4:**
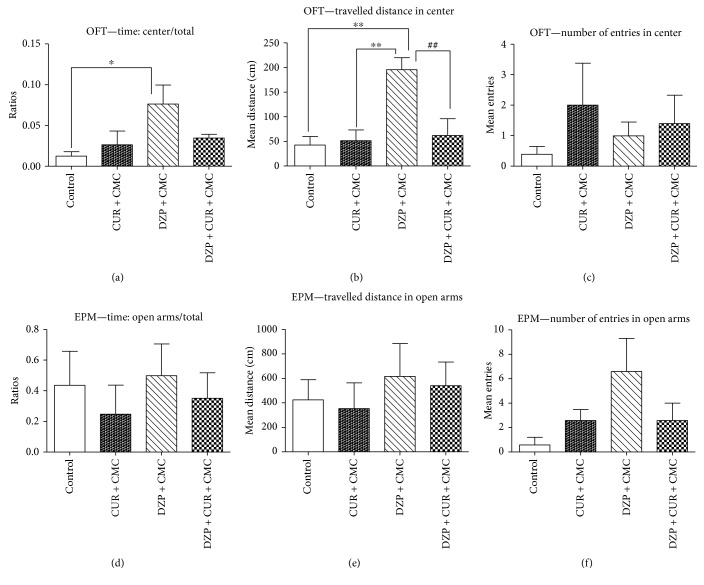
The effects of curcumin on emotionality in the open field test (OFT) (a, b, c) and in the elevated plus maze (EPM) (d, e, f). The center time/total time ratio (a) increased in the diazepam-treated group as compared to the control group (*p* < 0.05). The travelled distance in the center (b) enhanced in diazepam-treated rats as compared to the curcumin and control group (*p* < 0.01). The DZP + CUR + CMC group exhibited lower scores as compared to DZP + CMC (*p* < 0.01). The number of entries in the center (c) diminished in the diazepam group as compared to other groups, without significant statistical difference. The open arms/total time ratio (d) increased 1.41 times in DZP + CMC as compared to DZP + CUR + CMC. The travelled distance in open arms (e) increased 1.14 times in DZP + CMC as compared to DZP + CUR + CMC. The number of entries in open arms (f) increased 2.53 times in DZP + CMC as compared to DZP + CUR + CMC. The differences for emotionality (d, e, f) in the EPM were not statistically significant. Each group consisted of 5 rats. Results are expressed as mean ± SEM. ^∗^*p* < 0.05 as compared with control/CUR + CMC; ^∗∗^*p* < 0.01 as compared with control/CUR + CMC; and ^##^*p* < 0.01 as compared with DZP + CMC.

The influence of 28 days of curcumin administration on the emotionality, tested in the OFT and EPM, is exemplified in Figure 4.

Regarding the emotionality in the OFT, the diazepam-treated rats spent significantly more time the center of the open field arena as compared to the control group (*p* < 0.05), travelled a greater distance in the center, as compared to both the curcumin and the control groups (*p* < 0.01). Conversely, curcumin administration diminished the travelled distance in the center as compared to the diazepam group (DZP + CUR + CMC versus DZP + CMC, *p* < 0.01). Neither diazepam nor curcumin administration significantly influenced the central number entries of the rats.

The EPM test was designed to assess the anxiety-like behavior of rodents, as being considered the first-choice test for anxiolytic drugs screening and for the evaluation of anxiety in basic research [35]. Thus, more time spent, more travelled distance, and more entries made in the open arms of the EPM test apparatus during a 5 min test session, are indicative of low anxiety-like behavior. All three previous mentioned items tended to be increased in the diazepam group, but the differences for emotionality in the EPM were not statistically significant (DZP + CMC versus DZP + CUR + CMC, *p* > 0.05).

**Figure 5:**
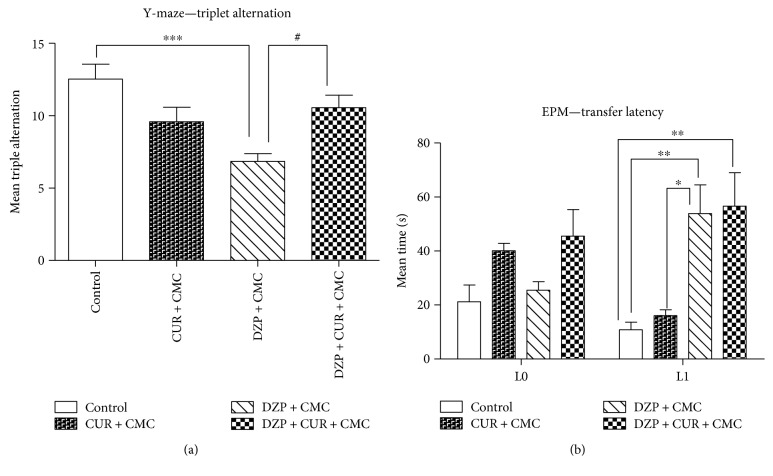
The effects of curcumin administration on memory in the Y-maze (a) and elevated plus maze (EPM) (b). The DZP group exhibited a significantly higher number of errors (a) in the Y-maze test as compared to the control group (*p* < 0.001). Conversely, curcumin administration significantly reversed the impairment of spontaneous alternation behavior (*p* < 0.05). Diazepam administration increased the transfer latency (b) both as compared to curcumin (*p* < 0.05) and as compared to the control group (*p* < 0.01) in L1. Each group consisted of 5 rats. Results are expressed as mean ± SEM. ^∗^*p* < 0.05 as compared with control/CUR + CMC; ^#^*p* < 0.05 as compared with DZP + CMC; ^∗∗^*p* < 0.01 as compared with control/CUR + CMC; and ^∗∗∗^*p* < 0.001 as compared with control/CUR + CMC.

The effects of curcumin administration on memory in the Y-maze (A) and elevated plus maze (EPM) (B) are illustrated in Figure 5. The Y-maze apparatus is used in order to assess the spatial working memory in rodents, based on the rodents desire to explore new environments. It counts the number of arm entries and the number of triple triads recorded in order to calculate the percentage of triple alternations [26]. Thus, the DZP group exhibited a significantly higher number of errors in the Y-maze test as compared to the control group (*p* < 0.001). Conversely, curcumin administration significantly reversed the impairment of spontaneous alternation behavior (DZP + CUR + CMC versus DZP + CMC; *p* < 0.05).

Nowadays, the EPM is also used to measure spatial long-term memory in mice and rats based on transfer latency scores [1, 3, 28, 29]. The transfer latency recorded by the Smart software, in the EPM, indicated that diazepam-treated rats displayed significantly higher latency scores both as compared to curcumin (*p* < 0.05) and as compared to the control group (*p* < 0.01) in L1.

### 3.2. Oxidative Stress Assessment in the Brain and Blood

**Figure 6:**
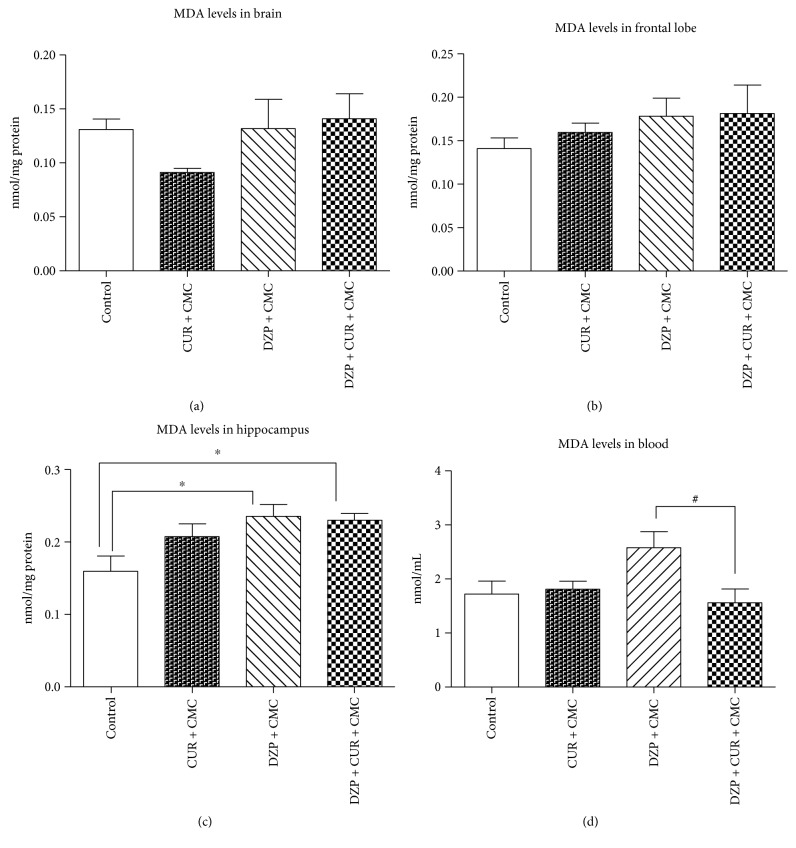
The effects of curcumin administration on the malondialdehyde (MDA) levels in different regions of the brain (a, b, c) and blood (d). The levels of MDA increased after DZP treatment, both in whole brain (a) homogenate and in frontal lobe (b), but without any statistical significance (DZP + CMC versus CUR + CMC, *p* > 0.05). MDA displayed higher levels in DZP + CMC versus control (*p* < 0.01) in the hippocampus (c). CUR administration diminished the same parameter in the blood (d) (*p* < 0.05). Each group consisted of 10 rats. Results are expressed as mean ± SEM. ^∗^*p* < 0.05 as compared with control/CUR + CMC; ^#^*p* < 0.05 as compared with DZP + CMC.

The malondialdehyde (MDA) levels in the brain and blood of rats pretreated with curcumin after DZP administration are illustrated in Figure 6.

The MDA levels increased after diazepam treatment, both in whole brain homogenate (0.13 ± 0.02 versus 0.09 ± 0.003 nmol/mg protein in CUR + CMC; *p* > 0.05) and in frontal lobe (0.17 ± 0.02 versus 0.15 ± 0.01 nmol/mg protein in the same group; *p* > 0.05) but without any statistical significance. MDA displayed higher levels in DZP + CMC (0.23 ± 0.01 nmol/mg protein) versus control (0.15 ± 0.02; *p* < 0.01) in the hippocampus. Curcumin administration significantly decreased the blood MDA level (1.56 ± 0.25 nmol/mL) in comparison to DZP-treated rats (2.57 ± 0.29 nmol/mL, *p* < 0.05).

**Figure 7:**
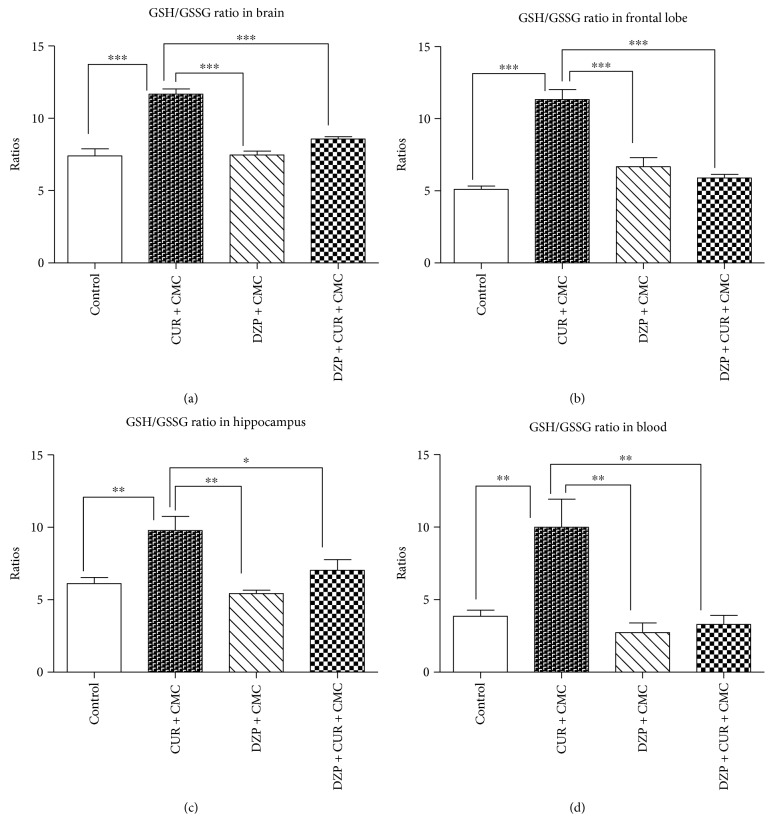
The effects of curcumin administration on the GSH/GSSG ratios in different regions of the brain (a, b, c) and blood (a). GSH/GSSG ratio was reduced in the diazepam group as compared to curcumin (*p* < 0.001) in whole brain (a), frontal lobe (b), hippocampus (c), and blood (d). GSH/GSSG ratio tended to increase in the DZP + CUR + CMC group as compared to DZP + CMC: 1.14 times in whole brain homogenate (a); 1.29 times in hippocampus (c); and 1.20 times in blood (d), but there was no statistical significance. Each group consisted of 10 rats. Results are expressed as mean ± SEM. ^∗^*p* < 0.05 as compared with control/CUR + CMC; ^∗∗^*p* < 0.01 as compared with control/CUR + CMC; and ^∗∗∗^*p* < 0.001 as compared with control/CUR + CMC.

The GSH/GSSG ratios in the brain and blood of rats from the experimental groups are illustrated in Figure 7. The GSH/GSSG ratio was significantly lower in the diazepam group as compared to curcumin in whole brain (7.48 ± 0.25 versus 11.66 ± 0.3738 nmol/mg protein; *p* < 0.001), frontal lobe (6.69 ± 0.61 versus 11.34 ± 0.68 nmol/mg protein, *p* < 0.001), hippocampus (5.44 ± 0.20 versus 9.77 ± 0.98 nmol/mg protein; *p* < 0.01), and blood (2.77 ± 0.62 versus 9.98 ± 1.94 nmol/mL; *p* < 0.01).

After 28 days of curcumin administration, the GSH/GSSG ratio tended to increase in whole brain homogenates (DZP + CUR + CMC: 8.58 ± 0.15 versus DZP + CMC: 7.48 ± 0.25 nmol/mg protein), hippocampus (7.04 ± 0.72 versus 5.44 ± 0.20 nmol/mg protein), and blood (3.33 ± 0.58 versus 2.77 ± 0.62 nmol/mL), but the differences were not statistically significant.

### 3.3. Quantitative Estimation of ERK 1/2 and NF-*κ*B/pNF-*κ*B and iNOS Expressions

**Figure 8:**
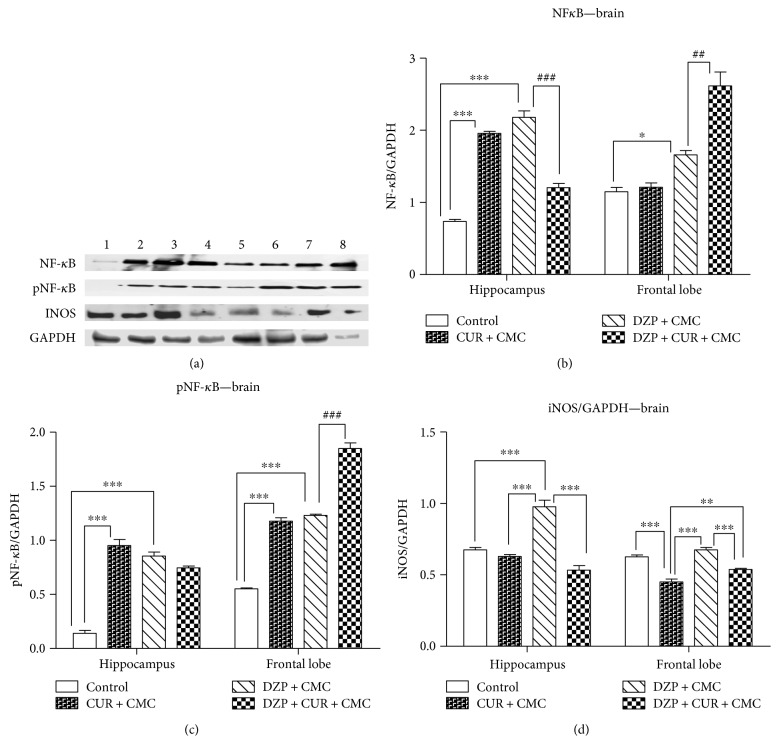
The effects of curcumin administration on the expression of NF-*κ*B, pNF-*κ*B, and iNOS in the brain. Expression of NF-*κ*B, pNF-*κ*B, and iNOS was analyzed by Western blot (WB). Image analysis of Western blot bands was done by densitometry, and results were normalized to GAPDH. WB images: 1–4 hippocampus, (1 = control, 2 = CUR + CMC, 3 = DZP + CMC, and 4 = DZP + CURC + CMC) and 5–8 frontal lobe (5 = control, 6 = CUR + CMC, 7 = DZP + CMC, and 8 = DZP + CURC + CMC); (*n* = 3). Each group consisted of 3 samples. ^∗^*p* < 0.05 as compared with control/CUR + CMC; ^∗∗^*p* < 0.01 as compared with control/CUR + CMC; ^∗∗∗^*p* < 0.001 as compared with control/CUR + CMC; ^##^*p* < 0.01 as compared with DZP + CMC; and ^###^*p* < 0.001 as compared with DZP + CMC.

The effects of curcumin administration on the expression of NF-*κ*B, pNF-*κ*B, and iNOS proteins in the brain are exemplified in Figure 8.

Either curcumin or diazepam alone significantly increased the expression of NF-*κ*B and pNF-*κ*B (the active form) as compared to the control group, in the hippocampus and frontal lobe (*p* < 0.001) as measured by Western blot. Both diazepam and curcumin administration significantly lowered the NF-*κ*B expression in the hippocampus (DZP + CUR + CMC versus DZP + CMC, *p* < 0.001). The pNF-*κ*B expression was lowered by both diazepam and curcumin, but without any statistical significance (*p* > 0.05). In the frontal lobe, there was a significant increase of both NF-*κ*B (*p* < 0.01) and pNF-*κ*B (*p* < 0.001) protein expressions after diazepam and curcumin administration (Figures 8(b) and 8(c)).

Diazepam administration significantly increased the expression of iNOS as compared to the control and curcumin group in the hippocampus (*p* < 0.001) and as compared to the curcumin group in the frontal lobe (*p* < 0.001) as measured by WB (Figure 8(d)). Both diazepam and curcumin administration significantly lowered the iNOS expression in the hippocampus and frontal lobe (*p* < 0.001). In the hippocampus, there was a significant decrease of iNOS in the curcumin group as compared to control (*p* < 0.001) (Figure 8(d)).

**Figure 9:**
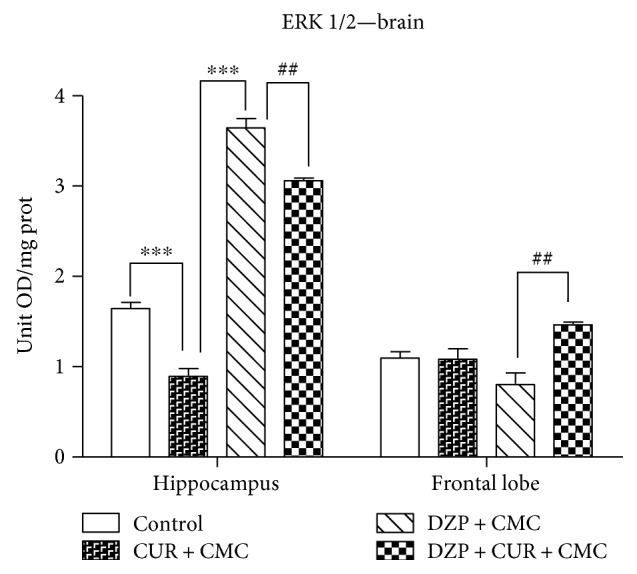
The effect of curcumin administration on the ERK 1/2 levels in the brain. In the hippocampus, ERK 1/2 level was significantly lower in the curcumin group as compared to control (*p* < 0.001). Diazepam administration significantly increased the ERK 1/2 protein as compared to curcumin (*p* < 0.001). Diazepam and curcumin administration diminished the ERK 1/2 level (*p* < 0.001). In the frontal lobe, diazepam and curcumin treatment stimulated the ERK 1/2 activation (*p* < 0.01). Each group consisted of 10 rats. Results are expressed as mean ± SEM. ^##^*p* < 0.01 as compared with DZP + CMC; and ^∗∗∗^*p* < 0.001 as compared with control/CUR + CMC.

The influence of curcumin administration on the ERK 1/2 level in the brain is shown in Figure 9.

In the hippocampus, ERK 1/2 levels were significantly lower in the curcumin group as compared to control (0.90 ± 0.13 versus 1.65 ± 0.10 OD/mg protein; *p* < 0.001). Diazepam administration significantly increased the ERK 1/2 protein level (3.64 ± 0.17 versus 0.90 ± 0.13 OD/mg protein *p* < 0.001). Diazepam and curcumin administration diminished the ERK 1/2 level (3.06 ± 0.04 versus 3.64 ± 0.17 OD/mg protein, *p* < 0.001). In the frontal lobe, diazepam and curcumin treatment stimulated the ERK 1/2 activation (1.47 ± 0.03 versus 0.81 ± 0.20 OD/mg protein, *p* < 0.01).

### 3.4. Histological Investigation and Immunohistochemical Analysis of iNOS

**Figure 10:**
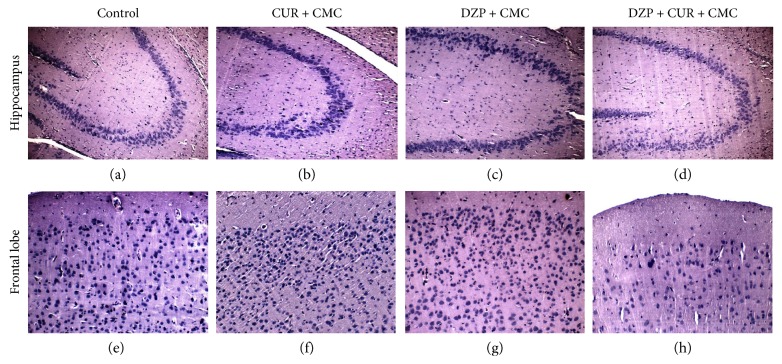
Representative photomicrographs of the hippocampus and frontal cortex of the four experimental groups. (a, b, c, d) showed the histological features of CA3 field in the hippocampus. (e, f, g, h) showed the frontal cortex micromorphology in the same experimental conditions. Magnification: ×200. H&E staining. Scale bar = 20 *μ*m.

The effects of curcumin administration on the micromorphology of the hippocampus and frontal cortex are illustrated in Figure 10. The brain tissue sections evaluated by morphometry did not show significant differences between the groups. Neither DZP nor curcumin influenced the morphology of the studied structures. The frontal cortex showed a normal architecture, and the distinct laminated organization of the cortex and the global morphology of the frontal neurons were intact.

**Figure 11:**
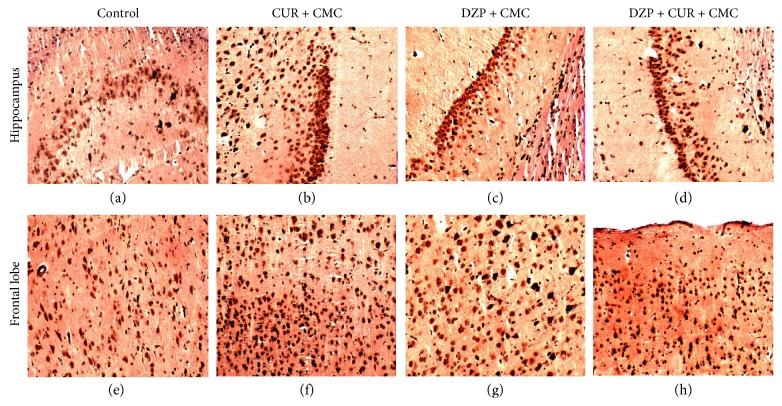
Immunohistochemical stain of iNOS in the hippocampus and frontal cortex of the four experimental groups. (a, b, c, d) showed the CA3 field in the hippocampus. (e, f, g, h) show the frontal cortex in the same experimental conditions.

Immunohistochemical expression of iNOS in the hippocampus and frontal cortex of the animals treated with DZP and curcumin is illustrated in Figure 11. DZP administration increased the iNOS expressions in the hippocampus and frontal lobe compared to the control group. Curcumin pretreatment in DZP-treated rats decreased the iNOS-positive cells in CA3 neurons and frontal lobe as compared to the DZP group.

## 4. Discussions

The results of the present study showed that 28 days of curcumin administration (150 mg/kg b.w.) significantly reduced the oxidative stress levels, both in the blood and in the brain tissue of the diazepam-treated rats. In the blood, curcumin diminished the lipid peroxidation (lower MDA levels) and improved the antioxidant activity (high GSH/GSSG ratio). Curcumin supplementation demonstrated an antioxidant activity by increasing the GSH/GSSG ratio and decreasing iNOS expressions in the hippocampus and frontal lobe. Regarding the behavioral effects of curcumin, there was an increase in general activity, revealed by significantly higher scores in the peripheral and total number of entries, assessed in OFT. Moreover, based on the Y-maze test results, CUR administration improved the spontaneous alternation behavior.

A wide variety of behavioral and pharmacological models is available for assessing cognitive functioning in rodent models, such as exteroceptive aversive stimulus models (e.g., behavior on mazes, passive avoidance, or active avoidance) and interoceptive aversive stimulus models (e.g., electroshock-induced amnesia, hypoxic stress-induced learning deficit, and pharmacological and discrimination assays) [36, 37]. In this particular study, we have chosen the interoceptive pharmacological behavioral model, such as diazepam administration, to simulate memory impairment [3, 16, 17] frequently associated with neurodegenerative disorders, traumatic head injury, dementia, and normal aging or exposure to continuous stress [4, 17].

In the present research, we provide evidence that DZP administration induced an increase of the oxidative stress parameters. Thus, we observed enhanced serum and hippocampus MDA levels and reduced GSH/GSSG ratios in whole brain, hippocampus, frontal lobe, and blood. These observations are consistent with the literature. Several studies reported enhanced TBARS levels and protein carbonyl content, as well as altered enzymatic activity, such as decreased glutathione reductase activity, in the cerebellum and brain stem [38, 39] after DZP administration. On the other hand, it has been shown that DZP pretreatment of acute stressed rats (immobilization) decreased the striatal lipid peroxidation levels and improved mitochondrial function [40].

The brain exhibits increased susceptibility to oxidative stress as it contains high concentrations of polyunsaturated fatty acids that are vulnerable to lipid peroxidation, and it also owes a modest antioxidant capacity. Moreover, the large oxygen consumption of the brain, the metabolism of catecholamines, and the release of neurotransmitters are considered important sources of free radicals, thus being associated with important oxidative damage [41, 42].

Regarding the behavioral tests, our findings reported an inhibitory effect of diazepam on the zone transition number and number of entries in the periphery, but with an antianxiety effect (increased center activity) on OFT. The rats exhibited a higher number of errors in Y-maze test and prolonged transfer latency in the EPM after diazepam treatment. The observed behavioral changes were also comparable with previous studies, which found that 2 mg/kg b.w. of diazepam did significantly reduce the latency to enter an open arm in an elevated T maze during the test session. The procedure is based on the avoidance of open spaces learned by the animals during training sessions [15]. Other authors also mentioned that pretreatment with diazepam (2.0–16.0 mg/kg i.p.) or scopolamine (3.0 mg/kg i.p.) produced impairment of anterograde memory, in a dose-dependent manner [43, 44]. Moreover, Kant et al. [45] revealed that administration of diazepam (0.5, 1.0, or 2.0 mg/kg, i.p.) prior to daily training on different mazes did not affect swim time, but it increased the swim errors, thus demonstrating that diazepam may affect the acquisition process and indicate memory impairment.

There are also clinical research data sustaining the amnestic effect of benzodiazepines [2, 46]. Considering all the aforementioned information, diazepam administration, in a dose-dependent manner, could be a suitable pharmacological model of memory impairment, therefore offering the possibility to study the efficiency of natural compounds in cognitive dysfunctions.

Recent studies suggested that oxidative stress, mitochondrial dysfunction, or inflammation plays an important role in memory decline [1, 3, 29]. So curcumin was chosen to be studied in this experimental model due to its various effects. Some authors explained the anti-inflammatory effects of curcumin by the inhibition of NF-*κ*B and ERK signaling pathways and by the reduction of the levels of IL-6, IL-1*β*, TNF-*α*, and iNOS in transgenic mouse brain [47, 48].

There is also scientific evidence that oxidative stress may consistently associate with memory impairment, especially in patients with neurodegenerative disorders. Andersen [8] wrote that the AD, PD, or amyotrophic lateral sclerosis (ALS) patients exhibited lower antioxidant enzymes levels in their brain and decreased GSH levels in the dopaminergic neurons in the substantia nigra, along with high MDA levels in the hippocampus, substantia nigra, or spinal fluid [8].

Based on the abovementioned data, some authors have proved the scavenging effects of curcumin, such as a glutathione concentration enhancer, thus preventing the lipid peroxidation [47, 49, 50]. So, indeed, we reached similar conclusions, regarding the antioxidant effect of curcumin, to these mentioned in the literature.

A possible explanation for the beneficial effect of curcumin on the spontaneous alternation behavior could be the antioxidant and the anti-inflammatory effect of this compound. The hippocampus is a brain structure involved in spatial navigation and memory [3], and the frontal lobe, responsible for conscious thought and decision-making, is related to short-term and long-term memory [51]. In consequence, we evaluated the effect of curcumin administration on diazepam-induced oxidative stress and memory impairment in rats by assessing the oxidative stress markers, along with the MDA levels in both blood and brain homogenates.

In addition, we evaluated the NF-*κ*B, pNF-*κ*B, and iNOS expressions and ERK 1/2 levels in the hippocampus and frontal lobe. The NF-*κ*B system is widely expressed in the central nervous system (CNS) [52]. The NF-*κ*B pathway can be activated by pathogen-associated molecules (lipopolysaccharide), multiple inflammatory (cytokines, chemokines), neurotransmitters, neurotrophic factors and neurotoxins, bacterial products, stress, and reactive oxygen species (ROS) [52–54]. In our study, one dose of DZP increased the NF-*κ*B and ERK 1/2 levels and the activation of NF-*κ*B, both in the hippocampus and in the frontal lobe. Curcumin diminished the expression and activation of both NF-*κ*B and pNF-*κ*B along with the ERK 1/2 levels and iNOS expression data, in the hippocampus of diazepam-treated rats, which are comparable with other reports. Accordingly, previous studies showed the inhibitory effect of curcumin on NF-*κ*B activity in different cell types [53, 55]. However, in the frontal lobe, our results were contradictory with the abovementioned data. Curcumin administration in diazepam-treated rats induced activation of NF-*κ*B and increased ERK 1/2 levels in the frontal lobe. These contradictory findings can be partially explained by regional heterogeneity regarding the density of receptors for benzodiazepines and differences in the accessibility of receptors to hydrophobic and hydrophilic ligands in the presence of oxidative stress [56]. It is known that lipid peroxidation of the membrane increases its viscosity and alters the coupling of the receptor to the effector system. Probably, the increase of lipid peroxidation, even insignificant, in the frontal lobe of the DZP + CUR + CMC group, induced alteration of membrane receptors and increased the expression of NF-*κ*B and ERK 1/2 and also the activation of NF-*κ*B. Several studies have shown that a single dose of diazepam increased or decreased oxidative damage depending on the analyzed cellular fraction, and this modulatory effect is region specific [39]. Another very interesting aspect is the effect of curcumin administration on the expression NF-*κ*B both in the hippocampus and in the frontal lobe and also on its activation in the hippocampus. Recent studies have demonstrated that NF-*κ*B was activated in the nervous system by physiological stimuli such as growth factors and glutamate and modulated synaptic plasticity and learning and long-term memory [57]. Moreover, it was noticed that the expression of various genes which have NF-*κ*B binding sites increased following learning [58] suggesting that the transcriptional targets of NF-*κ*B in the nervous system are important for plasticity and learning. It seems that NF-kΒ is critical for the neuroprotective adaptive responses following exposure to subthreshold noxious stimuli [59]. Unlike neurons, in glial cells, NF-*κ*B is present as an inactive complex with the I*κ*B proteins in the cytoplasm. This observation suggests that NF-*κ*B has different functions in the nervous system depending on the type of cell. However, the role of NF-*κ*B in the brain is not completely understood due to on one hand the difficulty of measuring transcription separately in neurons or glial cells and on the other hand due to a variety of NF-*κ*B responses depending on the strength of the triggering event. Therefore, more data are needed to further explain the pattern of NF-*κ*B activation and its role in the nervous system.

Histopathologically, no microscopic changes were found in all groups. Our data showed that the treated rats with a single administration of diazepam had no degree of histological brain damage, thus sustaining a functional dose-related damage rather than a morphological one. iNOS or NOS2 is an inducible enzyme involved in NO production and consequently in oxidative stress generation. In addition, recent studies have shown that NOS knockout was associated with behavioral changes and increased anxiolytic-like phenotype [60]. Therefore, the immunohistochemical and Western blot analysis of NOS2 expression in different regions of the brain and its relationship with lipid peroxidation was important to quantify the magnitude of oxidative stress. Our results showed that curcumin administration in DZP-treated rats decreased the immunohistochemical expression of iNOS in CA3 neurons and frontal lobe as compared to the DZP group and significantly lowered the iNOS expression in the hippocampus and frontal lobe as measured by Western blot.

In conclusion, our study demonstrates that curcumin administration (150 mg/kg b.w.) improved the spontaneous alternation behavior, decreased the oxidative stress levels, both in blood and brain tissue, diminished the activation of NF-*κ*B, pNF-*κ*B and lowered the MAPKs levels in the hippocampus and decreased the activation of iNOS in hippocampus and frontal lobe of the diazepam-treated rats. Thus, the improved GSH/GSSG ratio in the brain, along with the diminished activity of the NF-*κ*B, pNF-*κ*B, iNOS and low levels of MAPKs in the hippocampus, suggests that curcumin administration may improve the cognitive performance and offer brain protection.
